# Enhancing the Protective Immune Response to Administration of a LIVP-GFP Live Attenuated Vaccinia Virus to Mice

**DOI:** 10.3390/pathogens10030377

**Published:** 2021-03-21

**Authors:** Sergei N. Shchelkunov, Stanislav N. Yakubitskiy, Kseniya A. Titova, Stepan A. Pyankov, Alexander A. Sergeev

**Affiliations:** State Research Center of Virology and Biotechnology VECTOR, Rospotrebnadzor, Koltsovo, Novosibirsk 630559, Russia; yakubitskiy_sn@vector.nsc.ru (S.N.Y.); titova_ka@vector.nsc.ru (K.A.T.); pyankov_sa@vector.nsc.ru (S.A.P.); sergeev_ala@vector.nsc.ru (A.A.S.)

**Keywords:** poxviruses, vaccinia virus, vaccines, immune response, antibodies, protection

## Abstract

Following the WHO announcement of smallpox eradication, discontinuation of smallpox vaccination with vaccinia virus (VACV) was recommended. However, interest in VACV was soon renewed due to the opportunity of genetic engineering of the viral genome by directed insertion of foreign genes or introduction of mutations or deletions into selected viral genes. This genomic technology enabled production of stable attenuated VACV strains producing antigens of various infectious agents. Due to an increasing threat of human orthopoxvirus re-emergence, the development of safe highly immunogenic live orthopoxvirus vaccines using genetic engineering methods has been the challenge in recent years. In this study, we investigated an attenuated VACV LIVP-GFP (TK^-^) strain having an insertion of the green fluorescent protein gene into the viral thymidine kinase gene, which was generated on the basis of the LIVP (Lister-Institute for Viral Preparations) strain used in Russia as the first generation smallpox vaccine. We studied the effect of *A34R* gene modification and *A35R* gene deletion on the immunogenic and protective properties of the LIVP-GFP strain. The obtained data demonstrate that intradermal inoculation of the studied viruses induces higher production of VACV-specific antibodies compared to their levels after intranasal administration. Introduction of two point mutations into the *A34R* gene, which increase the yield of extracellular enveloped virions, and deletion of the *A35R* gene, the protein product of which inhibits presentation of antigens by MHC II, enhances protective potency of the created LIVP-TK^-^-A34R*-dA35R virus against secondary lethal orthopoxvirus infection of BALB/c mice even at an intradermal dose as low as 10^3^ plaque forming units (PFU)/mouse. This virus may be considered not only as a candidate attenuated live vaccine against smallpox and other human orthopoxvirus infections but also as a vector platform for development of safe multivalent live vaccines against other infectious diseases using genetic engineering methods.

## 1. Introduction

Preventive vaccination is the most reliable method to protect against viral infections. In this case, the highest efficiency is exhibited by live vaccines that are either weakly virulent human viruses closely related to pathogenic ones or pathogenic viral variants attenuated due to mutations/deletions in the viral genome [1].

For the long years of smallpox vaccination in the 19th and 20th centuries, there were used different strains of the virus that was classified as *Vaccinia virus* (VACV), a member of the *Orthopoxvirus* genus of the *Poxviridae* family [2,3]. The exact origin of these strains in most cases is not known, and they differ in pathogenicity towards different laboratory animals and in reactogenicity exhibited upon vaccination of humans [3,4,5,6]. During mass vaccination, all VACV strains caused a small percentage of severe adverse events, including encephalitis and encephalomyelitis, sometimes leading to death of vaccinees [5,7]. For this reason, following the global eradication of smallpox, the World Health Assembly adopted a resolution in 1980 [8], which called upon all countries to stop smallpox vaccination of the population.

Due to a 40-year cessation of smallpox vaccination, a huge part of the world’s population lacks immunity to both smallpox and any other zoonotic orthopoxvirus infections. This creates a new situation with circulation of zoonotic orthopoxviruses in the human population and, as a consequence, leads to changes in the ecology and range of sensitive hosts for different orthopoxvirus species [2]. Therefore, outbreaks of diseases caused by zoonotic orthopoxviruses, such as monkeypox virus, cowpox virus, or VACV, have been increasingly detected in people on different continents in recent years [9,10,11,12]. In this case, the classic live VACV vaccine is not suitable for protection against these infections because it can cause severe adverse events, in particular in people with a weakened immune system or immunodeficiencies (e.g., cancer patients, patients with organ transplantations, HIV-infected patients, etc.) [3,5,13]. In this regard, the development of modern safe vaccines against orthopoxvirus infections in humans and animals is topical importance [14].

The emergence of opportunities for genetic engineering of the VACV genome enabled the use of this virus as a molecular vector for development of multivalent vaccines against various infections and oncolytic VACV variants [3,6,15,16,17,18,19,20]. In this case, biological safety of generated recombinant VACVs has become the most important issue.

VACV attenuation is often achieved by targeted inactivation of certain viral genes and usually decreases the efficiency of VACV propagation in vivo. This may reduce the immune response to administration of an attenuated virus to patients at standard doses [3,10,13]. Therefore, it is important to search for viral genes whose modification may enhance the immunogenicity/protective potency of an attenuated VACV [1].

One of these candidate genes is the *A35R* gene of VACV, the product of which is an early intracellular protein that inhibits presentation of viral antigens by major histocompatibility complex class II (MHC II) molecules [21,22]. Deletion of the *A35R* gene was shown to increase production of virus-specific antibodies and enhance the protective potency of VACV [23,24].

VACV produces two infectious virion forms. The majority of viral progeny are intracellular mature virions (IMVs) that accumulate in an infected cell in large amounts and enter the environment only after destruction of the cell. A small part of the synthesized IMV particles are covered with an additional lipoprotein envelope, emerge on the cell surface at an early stage of the viral development cycle, and exist as cell-associated virions (CEVs). Some CEVs are detached from the cell surface and become free, the so-called extracellular enveloped virions (EEVs) [25]. In this case, EEVs ensure rapid spread of the virus throughout the body [25,26,27] and its effective penetration into cells [28].

A decrease in the efficiency of attenuated VACV variant propagation in vivo should lead to a proportional decrease in EEV production and, as a consequence, to a decrease in early dissemination of the virus throughout the body. We suggested that an increase in the yield of attenuated VACV EEVs may cause a more pronounced antiviral immune response. One of the promising research objects in this field is the *A34R* gene of VACV [27]. It encodes the A34 protein that is involved in the lipoprotein envelope of EEVs and controls the efficiency of their detachment from the infected cell surface and release in a free form into the intercellular space [27,29,30]. In most of the studied VACV strains during their replication in mammalian cell cultures, EEVs account for less than 1% of the viral progeny at an early stage of infection. Other virions occur in the cell as IMVs [25]. Multiple passages of VACV strain NYCBH in intracerebral infection of mice resulted in the generation of neurotropic VACV strain IHD-J [31] capable of producing EEVs in an amount of up to 30% of the total viral progeny and forming comet-like plaques on a monolayer of sensitive cells [26,27]. Differences in the amino acid sequence of protein A34 of another neurotropic laboratory VACV strain WR (forms EEVs in an amount of less than 1% of the infectious viral progeny in cell culture) from the analogous protein of VACV strain IHD-J were found to include only two point substitutions Asp110 → Asn and Lys151 → Glu [27]. Replacement of the *A34R* gene in VACV strain WR by a variant of this gene from the IHD-J strain significantly increases the yield of EEVs, which leads to more efficient dissemination of oncolytic VACV variants and improved anticancer activity of these viruses in vivo [32,33].

The study purpose was to investigate how introduction of two point mutations into the *A34R* gene, which increase the yield of EEVs, and deletion of the *A35R* gene, the protein product of which inhibits presentation of antigens by MHC II, affect the properties of attenuated VACV variant LIVP-GFP, which are associated with protection of LIVP-GFP-immunized mice against subsequent lethal orthopoxvirus infection.

## 2. Results

### 2.1. Creation of Recombinant Viruses

We used a clonal variant of VACV strain LIVP (Lister-Institute for Viral Preparations) that was produced earlier by three passages in cell culture by serial plaque-purifications using agarose overlay [34], and strain LIVP-GFP created from LIVP by insertion of the green fluorescent protein gene into the viral thymidine kinase gene (LIVP-TK^-^) [35].

The recombinant LIVP-GFP-A34R* strain was produced from VACV LIVP-GFP by transient dominant selection using the pMGCgpt-A34R* plasmid containing a mutant variant A34R* (Asp110 → Asn, Lys151 → Glu) of the *A34R* gene [36].

To introduce a deletion into the *A35R* gene of the LIVP-GFP-A34R* virus DNA, the pMGC20-gpt vector [34] was used to generate the pdA35R(A34R*) plasmid whose structure was confirmed by sequencing. This plasmid was used to generate the LIVP-GFP-A34R*-dA35R strain using transient dominant selection.

### 2.2. LIVP-GFP and LIVP-GFP-A34R* Viruses Exhibit Reduced Neurovirulence

**Figure 1:**
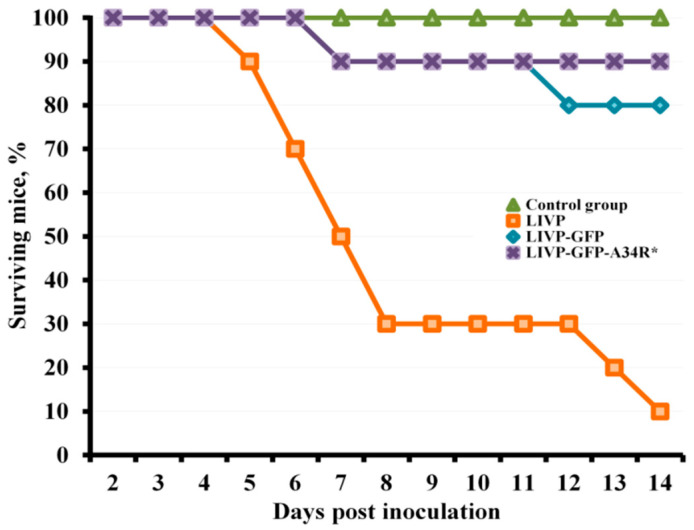
Changes in the death rate in newborn BALB/c mice intracerebrally inoculated with vaccinia virus (VACV) strains LIVP, LIVP-GFP, or LIVP-GFP-A34R* at a dose of 10 PFU or saline (Control group).

The ability of LIVP, LIVP-GFP, and LIVP-GFP-A34R* viruses to cause death of newborn mice upon intracerebral (i.c.) inoculation (dose of 10 plaque forming units (PFU)/10 μL/mouse) was studied in groups of 10 BALB/c mice, which were followed-up for 14 days after infection with an appropriate virus. By the experiment end, 90% of the VACV LIVP-infected mice died. Death of mice amounted to 20% for the LIVP-GFP strain and 10% for the LIVP-GFP-A34R* strain (Figure 1). In the control group of mice (i.c. inoculation of saline), all animals survived.

Deletion of the *A35R* gene is known to cause VACV attenuation [21]; therefore, neurovirulence of the LIVP-GFP-A34R*-dA35R strain was not additionally tested at this stage of the study.

### 2.3. Intradermal Injection of VACV LIVP Variants Provides Higher Production of Virus-Specific Antibodies Compared to Intranasal Administration

**Figure 2:**
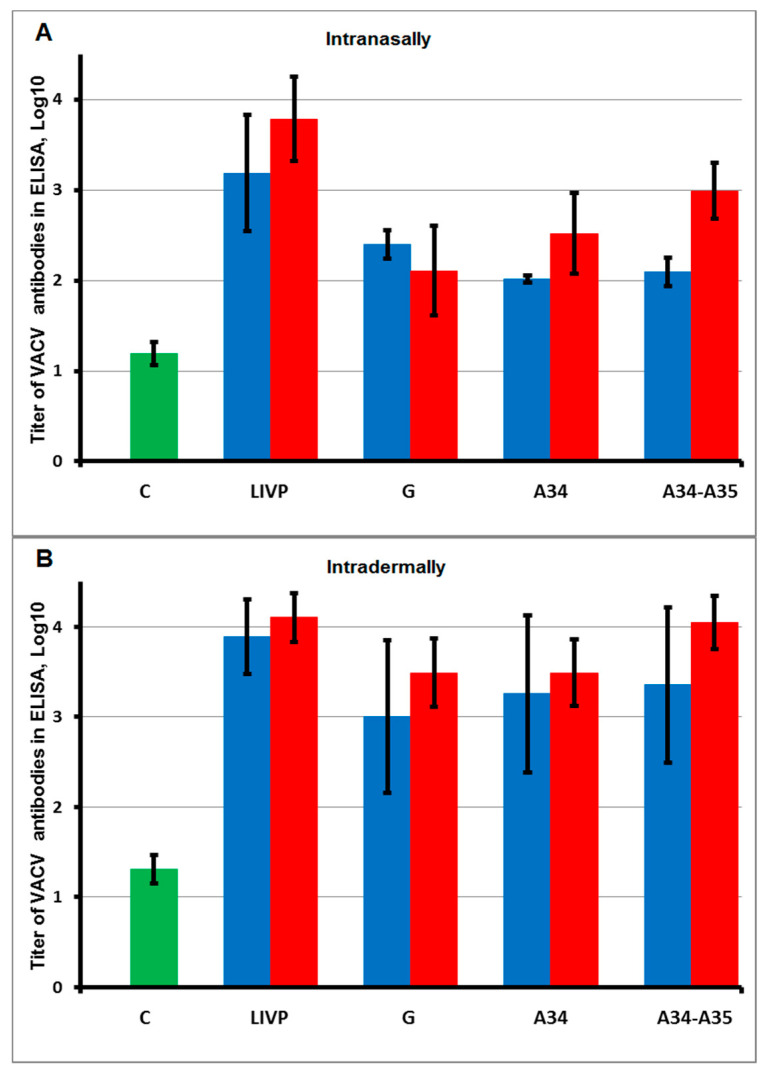
The concentration of VACV-specific IgG determined by ELISA in blood sera of mice intranasally (**A**) or intradermally (**B**) inoculated with LIVP, LIVP-GFP (G), LIVP-GFP-A34R* (A34), or LIVP-GFP-A34R*-dA35 (A34-A35) viruses at doses of 10^3^ PFU (blue bars) or 10^5^ PFU (red bars). C—control (blood sera of mice injected with saline).

BALB/c mice were intranasally (i.n.) or intradermally (i.d.) inoculated with LIVP, LIVP-GFP, LIVP-GFP-A34R*, or LIVP-GFP-A34R*-dA35R viruses at doses of 10^5^ or 10^3^ PFU/30 μL/animal (experimental groups). Animals in control groups were inoculated with saline instead of a virus. For both infective doses, no clinical manifestations of the disease and no weight loss in mice of any group were observed. Immunogenicity of the studied VACV variants was assessed by ELISA based on the level of VACV-specific IgG in mouse sera sampled 28 days after infection. The results of these assays, shown in Figure 2, demonstrate that i.d. immunization of mice with all LIVP variants provides higher antibody levels compared to their production after i.n. inoculation of the viruses.

### 2.4. Protection against Orthopoxvirus Infection Depends on the Route of Administration and the Dose of Viruses

Non-immunized BALB/c mice and mice 30 days post-inoculation with LIVP, LIVP-GFP, LIVP-GFP-A34R*, or LIVP-GFP-A34R*-dA35R viruses were i.n. infected with cowpox virus (CPXV) strain GRI-90 [37] at a dose of 56 LD_50_. The mice were individually weighed every 2 days, and were followed-up for survival for 14 days.

**Figure 3:**
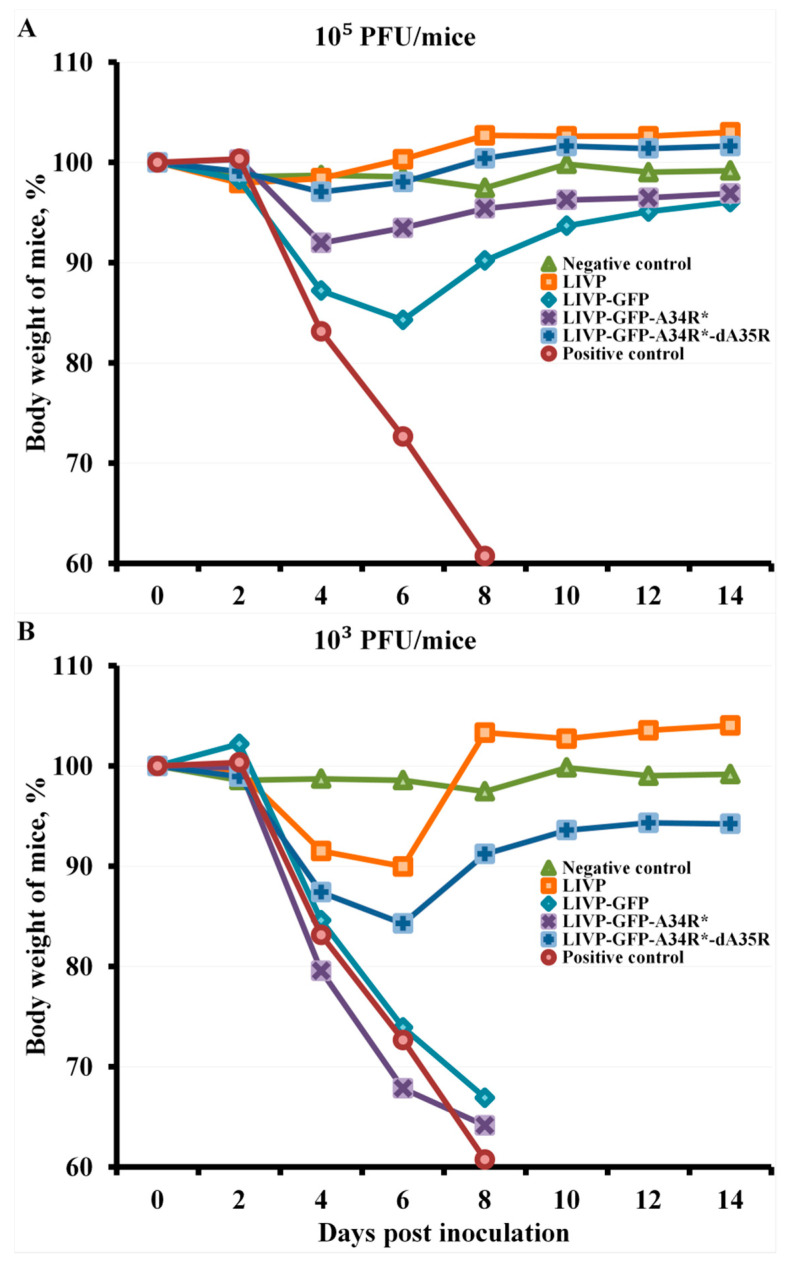
Changes in body weight of mice intranasally immunized with LIVP, LIVP-GFP, LIVP-GFP-A34R*, or LIVP-GFP-A34R*-dA35 viruses at doses of 10^5^ PFU (**A**) or 10^3^ PFU (**B**) after intranasal inoculation with CPXV GRI-90 at a dose of 56 LD_50_ on day 30.

At an infective dose of 10^5^ PFU for VACV variants, all mice, regardless of the route of viral administration, were completely protected against subsequent lethal CPXV infection, while all animals of the positive control group died on days 4–8. In this case, mice i.n. immunized with the LIVP-GFP virus had the highest temporary weight loss after infection with the CPXV virus. I.n. administration of the LIVP-GFP-A34R* mutant had a less pathogenic effect of CPXV infection on mice. The LIVP-GFP-A34R*-dA35R variant and the initial LIVP strain, at an immunization dose of 10^5^ PFU, ensured complete immunity of mice to CPXV infection (Figure 3A).

**Figure 4:**
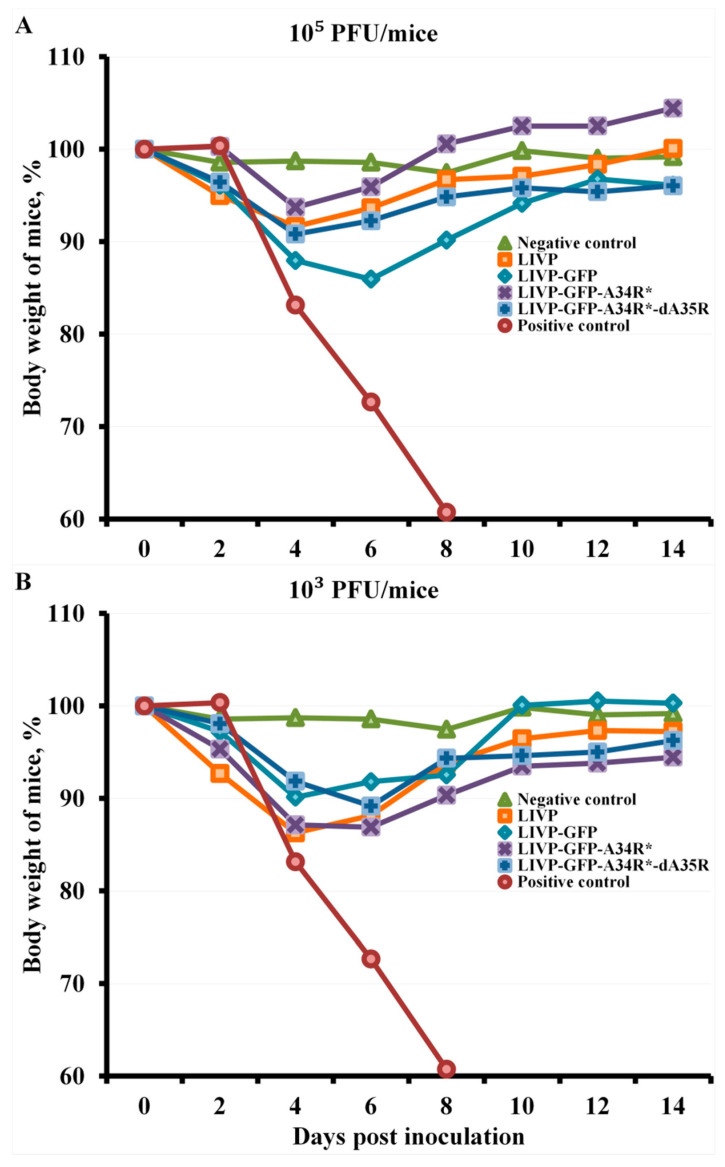
Changes in body weight of mice intradermally immunized with LIVP, LIVP-GFP, LIVP-GFP-A34R*, or LIVP-GFP-A34R*-dA35 viruses at doses of 10^5^ PFU (**A**) or 10^3^ PFU (**B**) after intranasal inoculation with CPXV GRI-90 at a dose of 56 LD_50_ on day 30.

After i.d. immunization of mice at a dose of 10^5^ PFU, their inoculation with the CPXV virus at a dose of 56 LD_50_ on day 30 of the experiment led to a slight pathogenic effect and a temporary decrease in body weight on days 4–6, with the greatest negative effect being observed in the group of mice immunized with the LIVP-GFP variant (Figure 4A).

**Figure 5:**
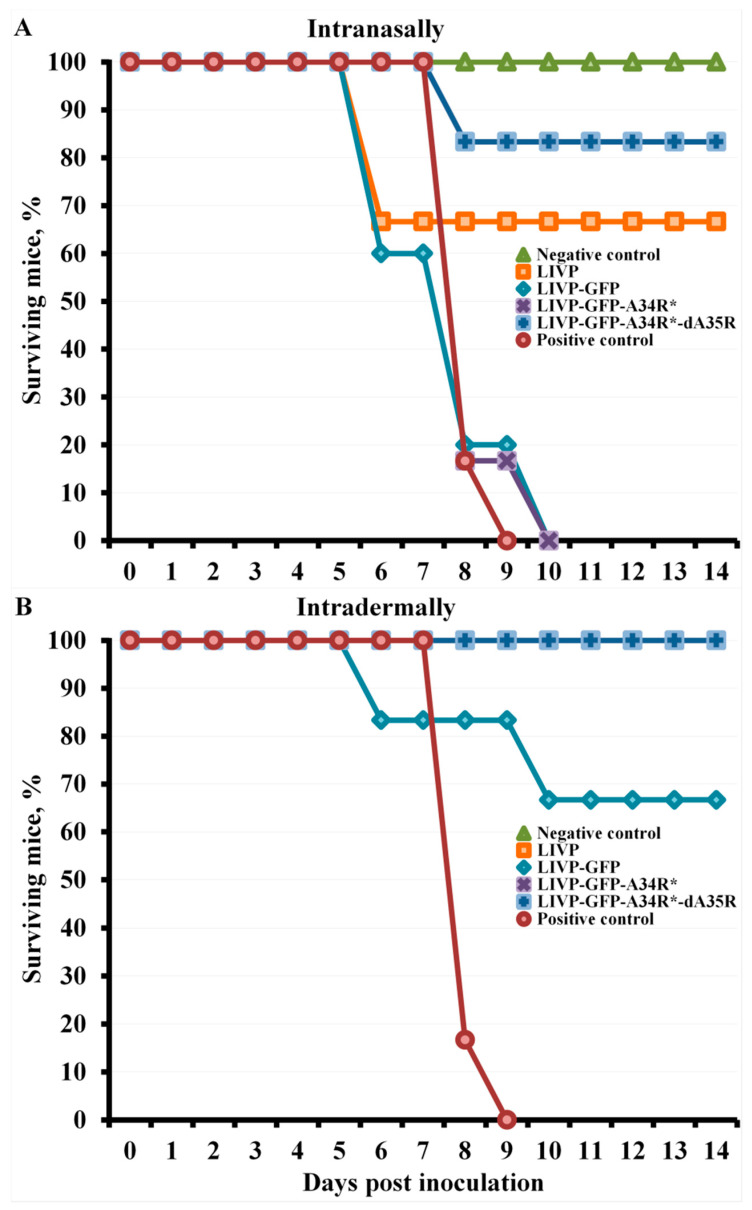
Changes in mortality of mice immunized intranasally (**A**) or intradermally (**B**) with LIVP, LIVP-GFP, LIVP-GFP-A34R*, or LIVP-GFP-A34R*-dA35R viruses at a dose of 10^3^ PFU/animal after their intranasal inoculation with CPXV GRI-90 at a dose of 56 LD_50_ on day 30 of the experiment.

Immunization at a dose of 10^3^ PFU did not provide complete protection against CPXV infection in some experimental groups of mice (Figure 5). I.d. immunization with all studied VACV LIVP variants, except LIVP-GFP, at this low dose ensured complete protection of mice against lethal CPXV infection (Figure 5B), but there was a temporary decrease in body weight of mice after CPXV infection in all experimental groups (Figure 4B).

The arithmetic means of mouse body weight are given for groups of 6 animals immunized with appropriate viruses as well as for non-immunized and not inoculated (Negative control) or inoculated with CPXV GRI-90 (Positive control) groups. Curves for negative control and mice immunized with LIVP are not significantly different within 8–14 days post inoculation.

I.n. immunization with VACV variants at a dose of 10^3^ PFU provided partial protection of mice against subsequent CPXV infection only in the case of LIVP (protection of 67% of animals) and LIVP-GFP-A34R*-dA35R (83%) strains (Figure 5A). In these groups, survived mice developed CPXV infection, but the animals’ condition normalized by day 8 (Figure 3B). In groups of mice i.n. immunized with VACV LIVP-GFP or LIVP-GFP-A34R* at a dose of 10^3^ PFU as well as in the positive control group, all animals died by day 8.

The arithmetic means of mouse body weight are given for groups of 6 animals immunized with appropriate viruses as well as for non-immunized and not inoculated (Negative control) or inoculated with CPXV GRI-90 (Positive control) groups.

Data are given for groups of 6 animals immunized with appropriate viruses as well as for non-immunized and not inoculated (Negative control) or inoculated with CPXV GRI-90 (Positive control) groups. (B) Curves for negative control and LIVP, LIVP-GFP-A34R*, and LIVP-GFP-A34R*-dA35R groups overlap.

## 3. Discussion

Vaccinia virus (VACV) was used as a live smallpox vaccine that, for the first time, allowed eradication of highly infectious disease in humans [4,7]. Despite the eradication of smallpox announced in 1980 and the recommendation to discontinue smallpox vaccination, interest in VACV was soon renewed due to the opportunity for genetic engineering of the genome of this virus using targeted insertion of foreign genes or deletion of certain viral genes. This genomic technology has enabled generation of stable attenuated VACV strains that produce antigens of various infectious agents [3]. In recent years, identification of the functions of numerous VACV genes and an increasing threat of human orthopoxvirus re-emergence have prompted the development of highly immunogenic live attenuated orthopoxvirus vaccines using genetic engineering methods [1,14].

Deletion or modification of multiple genes is an established strategy to improve attenuation of live organisms across different domains of life [38,39,40,41]. To date, modification or deletion of some VACV genes is known to increase the immunogenicity of this virus [1,10]. One of these examples is the VACV *A34R* gene that controls the release of EEVs from infected cells [25,27,42]. Laboratory VACV strains WR and IHD-J, which differ significantly in the level of EEV production, differ in the amino acid sequence of this protein only in two positions, 110 and 151. The C-terminal lectin-like domain of the viral A34 glycoprotein located on the surface of extracellular virions provides highly specific interaction between virions and cell surface carbohydrates. The Lys151 → Glu substitution in this domain of the A34 protein reduces the binding efficiency of VACV CEVs to the cell surface and increases the release of EEVs into the environment [27,29,43].

A VACV A34 glycoprotein segment between amino acid residues 80 and 130 is the region of interaction between virion proteins A34 and B5, and this complex of surface EEV proteins plays an important role in the binding of this form of virions to the cell surface [30]. The Asp110 → Asn mutation in the A34 glycoprotein affects its binding to the B5 protein and, probably, leads to an additional increase in the EEVs yield.

The considered *A34R* gene mutations increasing production of VACV EEVs do not reduce infectivity of the virus [44]. Additionally, EEVs are known to infect cells more efficiently than IMVs and differ in the mechanism of adsorption on the plasma membrane surface and penetration into the cell [28]. Recently, we demonstrated that introduction of these mutations into the *A34R* gene of VACV strain LIVP increases EEV production by the virus, decreases viral neurovirulence, and enhance production of VACV-specific antibodies upon i.d. injection of the virus to mice at high doses (10^8^–10^6^ PFU) [45].

Additionally, the VACV early intracellular protein A35 is known to inhibit presentation of viral antigens by MHC II [21,46]. Deletion of the *A35R* gene was experimentally shown to increase production of virus-specific antibodies and enhance the protective potency of VACV LIVP [23,24].

As an object of comparative studies, we selected VACV strain LIVP, which is used in Russia as a first generation smallpox vaccine, and an attenuated recombinant LIVP-GFP strain generated from VACV LIVP by insertion of the green fluorescent protein gene into the viral thymidine kinase gene. We studied how modification of the *A34R* gene and deletion of the *A35R* gene may affect the immunogenic and protective properties of the attenuated LIVP-GFP strain.

Because encephalitis and encephalomyelitis are the most serious adverse events of live VACV vaccination, neurovirulence of the produced VACV strains should be studied. The conventional method for assessing VACV neurotoxicity is intracerebral inoculation of suckling mice [47]. Our study has shown (Figure 1) that LIVP-GFP and LIVP-GFP-A34R* almost do not differ from each other in this parameter and exhibit significantly reduced neurovirulence compared to that of the parental LIVP strain.

Because deletion of the *A35R* gene attenuates VACV [21], neurovirulence of the LIVP-GFP-A34R*-dA35R strain was not additionally analyzed at this stage of the study.

Virus-specific antibodies induced by VACV immunization are known to play a key role in protecting animals against recurrent orthopoxvirus infection [1,13]. Therefore, the levels of VACV-specific antibodies induced by the studied viruses in mice were assessed by ELISA.

To elucidate how mutations in the *A34R* gene and deletion of the *A35R* gene affect the immunogenicity of VACV attenuated by inactivation of the viral thymidine kinase gene, levels of VACV-specific antibodies were determined by ELISA in blood sera sampled 28 days after i.n. or i.d. inoculation of mice with LIVP, LIVP-GFP, LIVP-GFP-A34R*, or LIVP-GFP-A34R*-dA35R viruses at doses of 10^5^ or 10^3^ PFU/mouse. The obtained data (Figure 2) demonstrate that i.d. inoculation of the studied viruses induced higher production of VACV-specific antibodies compared to their levels after i.n. administration. Apparently, this is due to the fact that the LIVP strain was produced by adapting the Lister strain to propagation on calf skin [4].

It should be noted that ELISA was used to detect IMV-specific IgG and we had no opportunity to analyze antibodies specific to EEV antigens. Therefore, the assessment of the protection against lethal orthopoxviral infection in this work was the main integrative indicator of the immunogenicity of the studied VACV strains.

All mice inoculated with VACV variants at an infective dose of 10^5^ PFU, regardless of the route of virus administration, were completely protected against subsequent CPXV infection at a dose of 56 LD_50_, while all animals in the positive control group died on days 4–8.

I.n. administration of the attenuated LIVP-GFP strain induced a significantly lower level of VACV-specific antibodies compared to that induced by the parental LIVP strain, and introduction of target mutations in the *A34R* gene resulted in a slight increase in the production of IMV-specific IgG in response to infection of mice with the LIVP-GFP-A34R* virus at a dose of 10^5^ PFU. I.n. immunization with the double-gene mutant LIVP-GFP-A34R*-dA35R at a dose of 10^5^ PFU induced a significantly higher level of virus-specific antibodies compared to that induced by the LIVP-GFP strain (Figure 2A). According to the body weight loss, the greatest pathogenic effect of CPXV infection was observed in mice i.n. immunized with the LIVP-GFP virus at this dose. A lower pathogenic effect of CPXV infection on mice was revealed upon i.n. administration of the LIVP-GFP-A34R* mutant (Figure 3A). The LIVP-GFP-A34R*-dA35R variant and the original LIVP strain at an immunization dose of 10^5^ PFU ensured complete protection of mice against subsequent CPXV infection. These data on protective potency correlate with the level of antibodies induced by viruses (Figure 2A and Figure 3A).

I.n. administration of all studied LIVP-GFP variants at an immunization dose of 10^3^ PFU provided a low production level of VACV-specific antibodies (Figure 2A). This led to the fact that LIVP-GFP and LIVP-GFP-A34R* viruses did not provide protection against 56 LD_50_ of CPXV GRI-90 (Figure 3B and Figure 5A). In this case, the parental LIVP strain protected 67% of mice from CPXV infection, which may be explained by a significantly higher level of virus-specific antibodies compared to that in mice infected with LIVP-GFP and LIVP-GFP-A34R*.

Despite low levels of induced VACV IMV-specific antibodies, the LIVP-GFP-A34R*-dA35R variant provided protection against CPXV infection in 83% of experimental animals (Figure 2A and Figure 5A). In this case, the protective potency against orthopoxvirus infection is apparently associated not only with production of antibodies to IMV antigens, the level of which was assessed (Figure 2A), but also with unanalyzed antibodies to EEV antigens as well as with the cellular immune response.

I.d. immunization with the attenuated LIVP-GFP strain at a dose of 10^3^ PFU protected only 67% of mice against subsequent lethal CPXV infection (Figure 5B). At the same time, strain LIVP and attenuated LIVP-GFP-A34R*, and LIVP-GFP-A34R*-dA35R mutant strains completely protected all animals against 56 LD_50_ of CPXV. In this case, a temporary weight loss in animals i.n. inoculated with CPXV occurred in all groups of experimental mice (Figure 4B).

Therefore, our findings suggest that increased production of EEVs by the attenuated LIVP-GFP strain, which is associated with targeted point mutations in the *A34R* gene, and deletion of the *A35R* gene, the protein product of which inhibits presentation of antigens by MHC II, increases the protective potency of this virus in mice against secondary lethal orthopoxvirus infection. This is most clearly manifested in animals immunized with a low dose of viruses.

Apparently, the studied mutant viruses increase the protective potency not only due to production of antibodies to VACV IMV proteins but also due to more efficient induction of other mechanisms of the development of humoral and cellular immune responses to both virion and non-virion viral proteins [10,13]. The possibility of reducing the immunization dose of attenuated VACV LIVP-TK^-^ with mutant changes in the *A34R* and *A35R* genes may, if necessary, significantly simplify production of large amounts of live vaccine doses for mass vaccination.

The LIVP-TK^-^-A34R*-dA35R virus may be considered not only as a candidate attenuated live vaccine against smallpox and other human orthopoxvirus infections but also as a vector platform for developing safe multivalent live vaccines against other infectious diseases using genetic engineering methods.

## 4. Materials and Methods

### 4.1. Viruses

We used cowpox virus (CPXV) strain GRI-90 [37], a clonal variant of VACV strain LIVP (Lister-Institute for Viral Preparations) [34], and strain LIVP-GFP created from this clonal variant of LIVP by insertion of the green fluorescent protein gene into the viral thymidine kinase gene (LIVP-TK^-^) [35].

The recombinant LIVP-GFP-A34R* and LIVP-GFP-A34R*-dA35R strains were produced from VACV LIVP-GFP by transient dominant selection using the recombinant plasmids pMGCgpt-A34R* [36] or pdA35R(A34R*) created on the basis of the pMGC20-gpt vector [34].

The viruses were grown and titrated in cell culture of the CV-1 African green monkey kidney cell line as described in [45].

### 4.2. Animals

In the study, we used BALB/c line mice received from the Animal Farm of SRC VB Vector. Experimental animals were fed with a standard diet and water ad libitum in compliance with the veterinary regulations and the requirements for humane care and use of animals in experimental studies. Animal studies and manipulations were approved by the Bioethics Committee of SRC VB Vector (Protocol No. 08-09.2020 of 25 September 2020).

LIVP, LIVP-GFP, LIVP-GFP-A34R*, and LIVP-GFP-A34R*-dA35R viral preparations or saline were i.n. or i.d. inoculated into male and female BALB/c mice aged 3–5-weeks and weighing 13–16 g as described in [48]. The infective dose was 10^5^ or 10^3^ PFU/30 μL/animal. Each experimental and control group consisted of 6 animals. Each experiment was performed independently twice and similar results were obtained.

### 4.3. Sampling Mouse Serum

Twenty-eight days after inoculation of VACV preparations (experimental groups) or saline (control groups), blood was sampled intravitally from the retro-orbital venous sinus of mice using disposable sterile capillaries. Serum was isolated from mouse blood by precipitating blood cells via centrifugation. Individual mouse serum samples were thermally inactivated at 56 °C for 30 min and stored at −20 °C.

### 4.4. Assessment of the Protective Potency in Immunized Mice

On day 30 of the experiment (the second day after blood sampling), virus-immunized and control animals were inoculated with CPXV GRI-90 at a dose of 56 LD_50_. Mice were individually weighed every 2 days. Animals were followed-up for survival for 14 days. The arithmetic mean mouse body weight in each group was calculated at every time point and expressed as a percentage of the initial weight as described in [48].

### 4.5. Assessment of Viral Neurovirulence

Groups of 2–3-day-old BALB/c suckling mice (10 animals each) were intracerebrally (i.c.) inoculated with LIVP, LIVP-GFP, or LIVP-GFP-A34R* strains at a dose of 10 PFU/10 μL/mouse. Animals of the control group were i.c. inoculated with 10 μL of saline. The mice were followed-up for survival for 14 days.

### 4.6. Assessment of Lethality of Cowpox Virus

BALB/c mice weighing 19–22 g (6 animals per group) were i.n. infected with 10-fold dilutions of the CPXV GRI-90 preparation; survival of mice in groups was monitored for 14 days, and the 50% lethal dose (LD_50_) was calculated using the Spearman-Karber method [49]. The number of CPXV GRI-90 virus lethal doses in the experiment on evaluation of the protective potency of studied VACV strains was additionally determined by i.n. inoculation (50 μL/mouse) of non-immunized mice (4 animals per dose) weighing 19–22 g with 10-fold dilutions (1/10, 1/100, 1/1000) of the CPXV GRI-90 dose (1.5 × 10^8^ PFU/mouse) administered to mice immunized with VACV variants. The administered dose calculated by the Spearman-Karber formula was 56 LD_50_.

### 4.7. Enzyme-Linked Immunosorbent Assay of Mouse Serum

The enzyme-linked immunosorbent assay (ELISA) of individual mouse sera was performed as described in [48]. Purified IMVs of VACV strain LIVP were used as an antigen. The geometric means of log reciprocal titer of VACV-specific IgG were determined for experimental groups, and the confidence intervals were calculated for a 95% matching between each sample and the total population.

### 4.8. Statistics

Statistical treatment of results was carried out with standard methods using the software package Statistica 6.0 (StatSoft, Tulsa, OK, USA), with assessment of significant differences (*p* < 0.05) for a 95% confidence level [50].

## Data Availability

All raw data are available and provided upon request.
